# Positive Association Between Family and Teachers’ Tobacco Use on the Smoking Behaviors of Iraqi Adolescents Attending Schools – A Cross Sectional Study Using the Global Youth Tobacco Survey

**DOI:** 10.1177/1179173X241283468

**Published:** 2024-09-18

**Authors:** Fatima Al-Binali, Soha R. Dargham, Ziyad R. Mahfoud

**Affiliations:** 1 Medical Education, 36579 Weill Cornell Medicine-Qatar, Doha, Qatar; 2 Division of Epidemiology, Department of Population Health Sciences, 36579 Weill Cornell Medicine-Qatar, Doha, NY, USA

**Keywords:** Tobacco, E-cigarette, middle east, youth, smoking

## Abstract

**Objective:** Adolescent tobacco and E-cigarette use have been rising globally in the past decade. Iraq’s post-war conflict and economic crises posed psychosocial and mental health trauma, contributing to increased vulnerability to drug and substance use among adolescents. This study looks to assess the potential relationship between current tobacco and E-cigarette smoking and exposure to smoking at home and school among Iraqi adolescents attending schools.

**Methods:** Data analysis for the 2019 Iraq Global Youth Tobacco Survey, a cross-sectional study completed by 2560 Iraqi adolescents aged 11 to 17 years old was conducted. The survey tool which includes demographic, tobacco use, knowledge and attitudes towards tobacco use questions is anonymous and self-administered using paper-based bubble sheets that are scannable. Current tobacco and E-cigarette use (defined as past 30-days) were the main outcomes. Univariate and multivariate logistic regression models were used to assess the relationship between the main outcomes and the following variables: participants’ demographics, exposure to smoking, attitude and knowledge scores.

**Results:** Current tobacco and E-cigarette smoking prevalence among Iraqi adolescents attending school were 14.9% (95% CI: 13.5%-16.4%) and 9.7% (95% CI: 8.6%-11.0%), respectively. Exposure to smoking at home was high among fathers (39.1%), mothers (13.9%), siblings smoke (23.9%), other individuals smoke (56.1%). The percentages of students who witnessed people smoking within school premises was 45.7% and observed teachers smoking in schools was 57.6%. The current use of tobacco smoking among Iraqi adolescents was significantly and positively associated with exposure to smoking by the father (AOR = 1.39; 95% CI: 1.05-1.85), mother (AOR = 1.84; 95% CI: 1.30-2.60), sibling (AOR = 3.50; 95% CI: 2.62-4.67), teacher (AOR = 1.48; 95% CI: 1.10-1.98), and people in school (AOR = 1.99; 95% CI: 1.57-2.53). Similarly, the current use of E-cigarettes was significantly associated with father smoking (AOR = 2.02; 95% CI: 1.29-3.16), sibling smoking (AOR = 3.09; 95% CI: 2.04-4.67), and people smoking in school (AOR2.02; 95% CI: 1.39-2.95).

**Conclusion:** Stricter policies need to be enforced to ensure safer school environments that do not expose adolescents to smoking habits of teachers or other students.

## Introduction

Tobacco use is one of the leading causes of preventable deaths, responsible for more than 7 million deaths per year worldwide, among which 890,000 deaths are attributable to second-hand smoking.^1^ Research has demonstrated that tobacco smoking increases the risk of heart disease, respiratory diseases, and cancer.^1^ Between 1990 and 2019, North Africa, the Middle East, and sub-Saharan Africa had the most significant relative increase in the number of smokers among both males and females.^2^

Adolescents have been the target of the tobacco industry over the years, with almost 90% of present smokers starting smoking before the age of 18,^3^ with at least one in every ten adolescents between the ages of 13 and 15 using tobacco products worldwide.^1^ Report findings from the WHO Global Health Observatory showed that the prevalence of adolescent students that currently smoke cigarettes around the world is 19.33%, with higher tobacco use among male adolescents.^4^

Adolescents go through a challenging transitional period between childhood and adulthood, involving biological, psychological, and social changes that play an essential role in their development^5,6^ and enabling sensation-seeking and risk-taking behaviors.^7^ These behaviors are shaped heavily by their surrounding environment and peer influence that either approve or discourage actions.^8^ Although adolescents are affected by peer behaviors and influence, parents and family members’ actions can also indirectly promote risky behaviors, acting as role models to the developing adolescent.^9,10^ Thus, adolescence is a vulnerable and risky period, and the behaviors initiated at this stage are more likely to be passed into adulthood.^7,8^

Iraq has been going through a challenging period of war conflicts and economic crises for the past three decades, traumatically affecting the Iraqi community, especially its younger youth population.^11,12^ The post-war conflict’s psychosocial and mental health trauma contributed to increased vulnerability to drug and substance use among the population.^12^ It is evident that there is a marked increase in rate of Iraqi adolescent tobacco smoking in the past decade. A previous analysis of Iraq GYTS data in 2012 that estimated the prevalence as 21.8%,^13^ while a recent study done in Erbil, Iraq in 2019 estimated the prevalence of cigarette smoking among adolescents to be 27.6%.^14^ This is in spite of the 2012 anti-smoking laws that prohibits smoking in public places and selling tobacco to those who are less than 18 years old. Research using the Global School-based Student Health Survey reported an overall prevalence of smoking cigarettes of 19.4% among teenagers (13-17 years) in the Eastern Mediterranean Region; 28.2% and 10.7% among boys and girls, respectively.^15^ Specifically among Iraqi teenagers, the overall prevalence of smoking was 18.6%; 25.2% and 9.9% among boys and girls, respectively.^15^ Not many studies looked at adolescents’ current E-cigarette use, especially in the middle east region. The rise in tobacco smoking rates among adolescents could be caused by low socioeconomic status post-war conflicts, accompanied with psychological factors and nicotine addiction.^12,16^

Strong social networks, family influence and respect to teachers are long term features of the culture in Iraq and the Middle East Region. Understating how such relationships influence the behavior of adolescent re-smoking is imperative. Against this background, we aim to assess the potential relationship between family and teachers’ smoking behaviors on that of Iraqi adolescents attending schools. We hypothesize that adolescents whose parents or teachers smoke will have higher rates of tobacco use.

## Methods

### Study design, sampling, and survey

Data analyses were conducted on the data from the Iraq Global Youth Tobacco Survey (GYTS) data for the year 2019. GYTS is a cross-sectional and nationally representative survey of students attending schools, conducted by the World Health Organization (WHO), and US Centers for Disease Control and Prevention (CDC). The survey, originally developed in English, was translated to Arabic and back translated to ensure it accuracy. It uses a global standardized methodology, employing a two-stage sample design. The inclusion criteria for school selection in the GYTS included both public and private schools in all geographic areas of Iraq. The inclusion criteria for class selection in the GYTS comprised of classes that generally encompass boys and girls 13-15 years of age.^17^ Schools are selected with a probability that is proportional to enrollment size in the first stage. Classes are then randomly selected in the second stage, granting all students eligibility to participate in the survey. More information concerning the GYTS questionnaire and methodology can be accessed under the WHO website.^18^

In Iraq, the cross-sectional survey was administered by the Ministry of Health and WHO to a total of 2560 adolescents. With such a sample size the study is able to estimate any prevalence to within a margin of error of at most ±2% using 95% confidence interval. The overall response rate was 89%. Necessary ethical approvals were obtained by the national authorities such as Ministries of Public Health and Education. Parental consent was obtained and students were informed that participation was voluntary and that by filling the questionnaire they were giving their assent.

The GYTS is a self-administered questionnaire that includes 43 core questions related demographics, socio-economic status, tobacco use, cessation, second-hand smoke, access and availability of tobacco products, media and advertisements, knowledge, and attitudes.^19^ In addition, countries can opt to ask about other forms of tobacco use, such as shisha use, smokeless tobacco, or E-cigarettes. The survey is anonymous and self-administered using paper-based bubble sheets that are scannable.

### Measures

For this study, there were two outcome variables: current use of any tobacco product and current use of E-cigarettes.

Current use of any tobacco product was defined using the GYTS questions such as “During the past 30 days, on how many days did you smoke cigarettes”, as well as the other tobacco products including shisha, pipe, cigars, hand-rolled tobacco, and smokeless tobacco. Participants who responded with >=1 day of smoking were considered current tobacco users, while responses with 0 days were considered not current tobacco smokers.

Current use of E-cigarettes was defined using the following question “During the past 30 days, on how many days did you use electronic cigarettes?” Similarly, responses with >=1 day were considered current E-cigarette smokers, while responses with 0 days were considered not current E-cigarette smokers.

The four exposure variables assessed in this study included exposure to smoking at home, exposure to smoking at school, participant’s attitudes toward tobacco use, and knowledge of tobacco use.

Questions regarding exposure smoking at home included the following four questions: 1) “How often do you see your father (stepfather or mother’s partner) smoking in your home?”, 2) “How often do you see your mother (stepmother or father’s partner) smoking in your home?”, 3) “How often do you see your brother/sister smoking in your home?”, and 4) How often do you see other people smoking in your home?”. Answers “about every day” and “sometimes” were coded as “yes” to questions 1 through 4, “never” was coded as “no”, and “don’t have/don’t see this person” were kept the same.

Questions regarding exposure to smoking at school included the following three questions: 1) “During the past 30 days, did you see anyone smoke inside the school building or in the school yard (inside school walls)?”, 2) “During school hours, how often do you see teachers smoking in the school building?”, and 3) “During school hours, how often do you see teachers smoking outdoors on school premises?”. Answers to question 1 were reported as “yes” and “no”. Questions 2 and 3 were combined into one exposure named “Any teachers smoking on school premises” with responses of “about every day” and “sometimes” coded as “yes”, “never” coded as “no”, and “don't know” kept the same.

Adolescents’ attitudes towards tobacco smoking were assessed using the following seven questions: 1) “Are you in favor of banning smoking inside enclosed public places (such as schools, shops, restaurants, markets, cars)?”, 2) “Are you in favor of banning smoking at outdoor public places (such as playgrounds, streets, gardens, parks)?”, 3) “Do you think tobacco advertising should be banned?”, 4) “Do you think the sale of tobacco products to minors should be banned?”, 5) “Do you believe that tobacco companies try to get young people under age 18 to use tobacco products?”, 6) “Do you think smoking tobacco helps people feel more comfortable or less comfortable at celebrations, parties, or in other social gatherings?”, and 7) “Do you think smoking shisha helps people feel more comfortable or less comfortable at celebrations, parties, or in other social gatherings?”. For questions 1 through 5, participants who responded with “yes”, “definitely yes”, and “probably yes” were given a score of 1, while participants who responded with “no”, “definitely no”, and “probably no” were given a score of 0. For questions 6 and 7, participants who responded with “less comfortable” were given a score of 1, while participants who responded with “more comfortable” and “no difference” were given a score of 0. Scores of all seven questions were summed to yield an attitude score between 0 and 7. A higher score indicated more conservative/negative attitudes towards tobacco use.

Adolescents’ knowledge about tobacco smoking was assessed using the following three questions: 1) “Do you think the smoke from other people's tobacco smoking is harmful to you?”, 2) “Once someone has started smoking tobacco, do you think it would be difficult for them to quit?”, and 3) “Do you think the smoke from other people's shisha smoking is harmful to you?”. Participants who responded with “yes”, “definitely yes”, and “probably yes” were given a score of 1, while participants who responded with “no”, “definitely no”, and “probably no” were given a score of 0. Scores of all three questions were summed to yield a knowledge score between 0 and 3. A higher score indicated more knowledge about tobacco use.

Demographic variables included age, sex, grade level, and weekly money to spend. Ages of study participants ranged from 11 to 17 years old; due to having very few patients in some categories, those in ages 11 to 13 were grouped and reported as “≤13 years old.” Weekly spending money was based on the question “During an average week, how much money do you have that you can spend on yourself, however you want?” and ranged from <3000 to >14,000 Iraq Dinar (IQD), equivalent to approximately <2.63 to >12.30 U.S. Dollars (USD) respectively using the conversion rate of 1 USD = 1138 IQD during the time of data collection.^20^ Due to small frequency in higher categories, those spending more than 10,000 IQD, equivalent to 8.79 USD, were grouped and reported as “more than 10,000 IQD.”

### Statistical analysis

Frequency distributions were used to summarize demographic variables such as age group and gender while means and standard deviations (SD) were used for numeric variables. The main outcomes were summarized using frequency distributions along with 95% confidence intervals using the Wald Method. Crude associations between demographic and exposure variables and the two main outcomes were estimated using univariate logistic regression. Covariates with *P*-values ≤0.2 in univariate regression analysis were included in the multivariable models. Odds ratios (OR) and adjusted odds ratios (AOR) with their 95% confidence intervals (CI) and *P*-values are presented. Significance was set at the 5% level. Data analyses were conducted using IBM-SPSS (version 26, Armonk, New York, USA).

### Ethical approval

This study uses publicly available anonymized GYTS data, thus it was not required to obtain a separate ethical approval from the authors’ institution.

## Results

A total of 2560 students participated in the 2019 Iraqi GYTS. The demographics of the study participants are detailed in Table 1. Briefly, 25.9% of participants were 13 years old or younger, 24.4% were 14 years old, 20.9% were 15 years old, and the rest were older than 16 years old. Majority of students were males (69.9%). First middle grade participants accounted for 44.4% of total participants, followed by third middle with a percentage of 32.2. About 11.9% of students did not get any weekly allowance, while 23.5% and 24.6% of students received less than 2.63 USD and between 2.63 to 4.39 USD, respectively.

**Table 1.** The Frequencies of Demographic Measures and Exposure to Smoking at Home and School Among 2560 Iraqi Adolescents Attending Schools.

		N	%
Demographics			
Age	13 years or younger	647	25.9
	14 years	608	24.4
	15 years	521	20.9
	16 years	337	13.5
	17 years or older	383	15.3
Sex	Male	1771	69.9
	Female	762	30.1
Grade	First middle	1121	44.4
	Second middle	591	23.4
	Third middle	812	32.2
Weekly allowance	Don’t have any spending money	299	11.9
	Less than 2.63 USD	592	23.5
	2.63 - 4.39 USD	619	24.6
	4.39 - 6.15 USD	418	16.6
	6.15 - 8.79 USD	249	9.9
	More than 8.79 USD	343	13.6
Exposure to smoking at home and at school			
Father smoking	Yes	978	39.1
	No	823	32.9
	Don’t have/Don’t see this person	699	28.0
Mother smoking	Yes	350	13.9
	No	1413	56.0
	Don’t have/Don’t see this person	761	30.2
Siblings smoking	Yes	601	23.9
	No	1210	48.1
	Don’t have/Don’t see this person	706	28.0
Other people smoking	Yes	1395	56.1
	No	497	20.0
	Don’t have/Don’t see this person	596	24.0
People smoke in school	Yes	1131	45.7
	No	1344	54.3
Teachers smoking in school premises	Yes	1443	57.6
	No	772	30.8
	Don’t know	290	11.6

Table 1 also reports the frequencies of adolescents’ exposure to second-hand smoking at home and school and the prevalence of current use of any type of tobacco and E-cigarettes. Around 40% of the students reported seeing their fathers smoke, while 13.9% reported seeing their mothers smoke. About a quarter of students stated their siblings smoke (23.9%), and more than half of reported other individuals smoke at home (56.1%). The percentage of students who witnessed people smoking within school premises was 45.7%, and more than half of students observed teachers smoking in schools (57.6%).

Table 2 presents the estimated prevalence of current use of any type of tobacco and E-cigarettes. Prevalence of cigarette smoking was the highest among other types of tobacco with a rate of 14.9% (95% CI: 13.5%-16.4%), followed by cigar use of 13.0% (95% CI: 11.8%-14.4%), and shisha use of 11.9% (95% CI: 10.7%-13.3%). The prevalence of any tobacco smoking among participants was 30.3% (95% CI: 28.5%-32.1%), while that of E-cigarette smoking was 9.7% (95% CI: 8.6%-11.0%).

**Table 2.** Prevalence Estimates of Current Use of Any Type of Tobacco and E-Cigarettes Among 2560 Surveyed Iraqi Adolescents Attending Schools.

	n/N	% (95% CI)
Type of tobacco use in past 30 days		
Cigarette	358/2403	14.9 (13.5-16.4)
Shisha	276/2314	11.9 (10.7-13.3)
Pipe	225/2521	8.9 (7.9-10.1)
Cigar	329/2526	13.0 (11.8-14.4)
Hand-rolled tobacco	230/2516	9.1 (8.1-10.3)
Smokeless tobacco	265/2533	10.5 (9.3-11.7)
Any tobacco	774/2558	30.3 (28.5-32.1)
E-cigarettes	236/2424	9.7 (8.6-11.0)

CI: Confidence Interval.

Measures of the adolescents’ attitudes and knowledge toward smoking are presented in Table 3. A great percentage of students agreed on banning smoking inside and outside public places and banning tobacco advertisements and sales to minors. Around 70% of students reported that smoking tobacco or shisha makes people more comfortable or does not affect comfort at social gatherings. Participants in the study had an average score of 2.88 (SD: 1.66; range: 1-7) on the tobacco attitudes scale, with a median of 4.00. More than two-thirds of students agreed that the smoke from other people’s tobacco or shisha usage was harmful to them, while more than half of students reported that quitting tobacco would not be difficult. Participants had an average total score of 1.83 (SD: 0.99; range: 1-3) on the tobacco knowledge scale, with a median of 2.00.

**Table 3.** Attitudes and Knowledge Towards Tobacco Use Among 2560 Iraqi Adolescents Attending Schools.

		n	%
Adolescents’ attitudes toward tobacco use			
Are you in favor of banning smoking inside enclosed public places?	Yes	1907	77.6
	No	551	22.4
Are you in favor of banning smoking at outdoor public places?	Yes	1703	68.7
	No	776	31.3
Do you think tobacco advertising should be banned?	Yes	1381	59.5
	No	941	40.5
Do you think the sale of tobacco products to minors should be banned?	Yes	1823	76.1
	No	574	23.9
Tobacco companies try to get young people to use tobacco	Yes	1661	68.9
	No	749	31.1
Smoking tobacco makes people more comfortable at social gatherings?	Less comfortable	724	29.5
	More comfortable/No difference	1727	70.5
Smoking shisha makes people more comfortable at social gatherings?	Less comfortable	709	29.2
	More comfortable/No difference	1718	70.8
Total score	Mean ± SD	2.88 ± 1.66	
	Median (IQR)	4.00 (3.00-5.00)	
	Range	1.00-7.00	
Adolescents’ knowledge towards tobacco use			
Do you think the smoke from other people’s tobacco smoking is harmful to you?	Yes	1902	75.9
	No	604	24.1
Do you think the smoke from other people’s shisha smoking is harmful to you?	Yes	1669	66.4
	No	840	33.6
Do you think it would be difficult to quit smoking?	Yes	1116	44.2
	No	1407	55.8
Total score	Mean ± SD	1.83 ± 0.99	
	Median (Q1-Q3)	2.00 (1.00-3.00)	
	Range	1.00-3.00	

SD: standard deviation; Q1 and Q3 are the 25th and 75th percentiles.

The results of the multivariable analysis assessing the association between exposure to smoking and the current use of any tobacco product, adjusting for demographic factors, attitude and knowledge sum scores towards smoking are reported in Table 4. The odds of current use of tobacco use were significantly lower among the 13 years and younger students, as well as those who were 14 and 15 years old compared to students who were 17 years or older. Male adolescents had significantly higher odds of tobacco use as compared with females (AOR = 1.39; 95% CI: 1.02-1.88). Adolescents who did not receive spending money were significantly associated with lower tobacco use compared to adolescents who received more than USD8.79 spending allowance (AOR = 0.46; 95% CI: 0.29-0.71).

**Table 4.** Univariate and Multivariate Logistic Regression Analyses Assessing the Association Between Exposure to Smoking and Any Current Tobacco Use, Adjusting for Demographic Factors, Attitude and Knowledge Sum Scores Towards Tobacco Smoking.

		Any Current Tobacco Use		Univariate Analysis		Multivariate Analysis	
		Yes	No				
		n (%)	n (%)	OR (95% CI)	*P*-value	AOR (95% CI)	*P*-value
Age	13 years or younger	136 (21.0)	511 (79.0)	0.26 (0.19-0.34)	<0.001*	0.39 (0.25-0.60)	<0.001*
	14 years	143 (23.5)	465 (76.5)	0.30 (0.22-0.39)	<0.001*	0.53 (0.35-0.80)	0.002*
	15 years	127 (24.4)	394 (75.6)	0.31 (0.23-0.41)	<0.001*	0.38 (0.26-0.55)	<0.001*
	16 years	136 (40.4)	201 (59.6)	0.65 (0.48-0.87)	0.004*	0.82 (0.56-1.19)	0.288
	17 years or older	195 (51.0)	187 (49.0)	Ref.		Ref.	
Sex	Male	628 (35.5)	1141 (64.5)	2.53 (2.06-3.12)	<0.001*	1.39 (1.02-1.88)	0.035*
	Female	136 (17.8)	626 (82.2)	Ref.		Ref.	
Grade	First middle	314 (28.0)	806 (72.0)	0.60 (0.49-0.72)	<0.001*	0.88 (0.64-1.22)	0.457
	Second middle	120 (20.3)	470 (79.7)	0.39 (0.31-0.50)	<0.001*	0.51 (0.35-0.72)	<0.001*
	Third middle	321 (39.5)	491 (60.5)	Ref.		Ref.	
Weekly allowance	Don’t have any spending money	82 (27.4)	217 (72.6)	0.42 (0.30-0.59)	<0.001*	0.46 (0.3-0.71)	<0.001*
	Less than 2.63 USD	132 (22.3)	459 (77.7)	0.32 (0.24-0.43)	<0.001*	0.46 (0.32-0.66)	<0.001*
	2.63 - 4.39 USD	176 (28.4)	443 (71.6)	0.44 (0.34-0.58)	<0.001*	0.73 (0.51-1.04)	0.079
	4.39 - 6.15 USD	117 (28.0)	301 (72.0)	0.43 (0.32-0.58)	<0.001*	0.65 (0.44-0.96)	0.029*
	6.15 - 8.79 USD	87 (34.9)	162 (72.0)	0.60 (0.43-0.84)	0.003*	0.94 (0.62-1.43)	0.777
	More than 8.79 USD	162 (47.4)	180 (52.6)	Ref.		Ref.	
Father smoking	Don’t have	211 (30.2)	487 (69.8)	1.70 (1.35-2.15)	<0.001*	0.99 (0.69-1.44)	0.976
	Yes	364 (37.3)	613 (62.7)	2.33 (1.88-2.89)	<0.001*	1.39 (1.05-1.85)	0.024*
	No	167 (20.3)	656 (79.7)	Ref.		Ref.	
Mother smoking	Don’t have	250 (32.9)	510 (67.1)	1.69 (1.39-2.05)	<0.001*	1.22 (0.86-1.74)	0.259
	Yes	183 (52.3)	167 (47.7)	3.77 (2.96-4.82)	<0.001*	1.84 (1.30-2.60)	<0.001*
	No	318 (22.5)	1095 (77.5)	Ref.		Ref.	
Sibling smoking	Don’t have	194 (27.5)	512 (72.5)	1.63 (1.31-2.03)	<0.001*	1.00 (0.77-1.54)	0.629
	Yes	326 (54.2)	275 (45.8)	5.11 (4.12-6.34)	<0.001*	3.50 (2.62-4.67)	<0.001*
	No	228 (18.8)	982 (81.2)	Ref.		Ref.	
Other family smoking	Don’t have	166 (27.9)	430 (72.1)	1.77 (1.32-2.36)	<0.001*	1.13 (0.76-1.68)	0.551
	Yes	484 (34.7)	911 (65.3)	2.43 (1.88-3.13)	<0.001*	1.39 (0.99 -1.93)	0.051
	No	89 (17.9)	407 (82.1)	Ref.		Ref.	0.085
People smoke in school	Yes	438 (38.8)	692 (61.2)	2.33 (1.95-2.78)	<0.001*	1.99 (1.57-2.53)	<0.001*
	No	287 (21.4)	1057 (78.6)	Ref.		Ref.	
Teacher smoking	Don’t know	50 (17.2)	240 (82.8)	0.95 (0.67-1.35)	0.772	0.72 (0.47-1.10)	0.127
	Yes	565 (39.2)	878 (60.8)	2.93 (2.37-3.62)	<0.001*	1.48 (1.10-1.98)	0.009*
	No	139 (18.0)	633 (82.0)	Ref.		Ref.	
Attitude sum score; mean (SD)		3.44 (1.66)	4.07 (1.63)	0.79 (0.75-0.84)	<0.001*	0.81 (0.76-0.87)	<0.001*
Knowledge sum score; mean (SD)		1.61 (1.03)	1.93 (0.96)	0.73 (0.67-0.79)	<0.001*	0.81 (0.72-0.91)	<0.001*

SD: standard deviation; OR: odds ratio; AOR: adjusted odds ratio; CI: confidence interval.

Exposure to family members’ smoking habits was highly associated with increased susceptibility to tobacco smoking. Compared to adolescents with non-smoking fathers and mothers, adolescents with fathers and mothers who smoke had significantly higher odds of using tobacco products with an AOR of 1.39 (95% CI: 1.05-1.85) and 1.84 (95% CI: 1.30-2.60) respectively. Additionally, adolescents with siblings who smoke tobacco had significantly higher odds of tobacco smoking, 3.50 (95% CI: 2.62-4.67) times that of adolescents with non-smoking siblings. Students who observed people and teachers smoking within school premises were significantly associated with increased tobacco use compared to students who did not, with an AOR of 1.99 (95% CI: 1.57-2.53) for other people smoking in school and an AOR of 1.48 (95% CI: 1.10-1.98) for observing teachers smoking. Students with lower attitude (AOR = 0.81; 95% CI: 0.76-0.87) and knowledge (AOR = 0.81; 95% CI; 0.72-0.91) sum scores were significantly correlated with the current use of any tobacco product.

Similarly, the current use of E-cigarettes among adolescents attending school in Iraq was significantly associated with male sex (AOR = 2.02; 95% CI: 1.19-3.45), with higher weekly allowance (*P*-value <0.05), father smoking (AOR = 2.02; 95% CI: 1.29-3.16), sibling smoking (AOR = 3.09; 95% CI: 2.04-4.67), people smoking in school (AOR2.02; 95% CI: 1.39-2.95), and lower attitudes towards smoking (AOR = 0.76; 95% CI: 0.69-0.85). More details are included in Table 5.

**Table 5.** Univariate and Multivariate Logistic Regression Analyses Assessing the Association Between Exposure to Smoking and Current E-Cigarettes Use, Adjusting for Demographic Factors, Attitude and Knowledge Sum Scores Towards E-Cigarettes Smoking.

		Current E-Cigarettes Use		Univariate Analysis		Multivariable Analysis	
		Yes	No				
		n (%)	n (%)	OR (95% CI)	*P*-value	AOR (95% CI)	*P*-value
Age	13 years or younger	37 (5.9)	585 (94.1)	0.31 (0.20-0.48)	<0.001*	0.77 (0.39-1.52)	0.447
	14 years	49 (8.4)	532 (91.6)	0.45 (0.30-0.68)	<0.001*	0.88 (0.48-1.64)	0.693
	15 years	43 (8.7)	453 (91.3)	0.47 (0.31-0.71)	<0.001*	0.76 (0.44-1.32)	0.329
	16 years	34 (10.8)	280 (89.2)	0.60 (0.38-0.94)	0.026	0.80 (0.45-1.43)	0.456
	17 years or older	60 (16.9)	296 (83.1)	Ref.		Ref.	
Sex	Male	205 (12.3)	1462 (87.7)	3.41 (2.29-5.09)	<0.001*	2.02 (1.19-3.45)	0.010*
	Female	29 (3.9)	706 (96.1)	Ref.		Ref.	
Grade	First middle	83 (7.9)	972 (92.1)	0.60 (0.44-0.81)	<0.001*	0.88 (0.52-1.47)	0.618
	Second middle	53 (9.4)	511 (90.6)	0.73 (0.51-1.03)	0.075	1.29 (0.76-2.20)	0.344
	Third middle	97 (12.5)	678 (87.5)	Ref.		Ref.	
Weekly allowance	Don’t have any spending money	23 (8.3)	254 (91.7)	0.33 (0.20-0.54)	<0.001*	0.38 (0.20-0.73)	0.004*
	Less than 2.63 USD	31 (5.5)	537 (94.5)	0.21 (0.13-0.33)	<0.001*	0.28 (0.16-0.50)	<0.001*
	2.63 - 4.39 USD	40 (6.8)	549 (93.2)	0.26 (0.17-0.40)	<0.001*	0.36 (0.22-0.60)	<0.001*
	4.39 - 6.15 USD	31 (7.8)	368 (92.2)	0.30 (0.19-0.48)	<0.001*	0.46 (0.27-0.79)	0.005*
	6.15 - 8.79 USD	36 (15.1)	202 (84.9)	0.64 (0.41-0.99)	0.049	0.77 (0.44-1.35)	0.361
	More than 8.79 USD	69 (21.8)	248 (78.2)	Ref.		Ref.	
Father smoking	Don’t have	44 (6.8)	599 (93.2)	1.20 (0.78-1.84)	0.408	1.52 (0.84-2.75)	0.169
	Yes	133 (14.2)	802 (85.8)	2.70 (1.91-3.84)	<0.001*	2.02 (1.29-3.16)	0.002*
	No	46 (5.8)	750 (94.2)	Ref.		Ref.	
Mother smoking	Don’t have	58 (8.3)	642 (91.7)	1.03 (0.74-1.44)	0.859	0.82 (0.49-1.38)	0.459
	Yes	56 (17.3)	268 (82.7)	2.38 (1.68-3.37)	<0.001*	1.10 (0.67-1.80)	0.698
	No	111 (8.1)	1266 (91.9)	Ref.		Ref.	
Sibling smoking	Don’t have	32 (4.9)	623 (95.1)	0.74 (0.48-1.13)	0.157	0.62 (0.34-1.12)	0.115
	Yes	116 (20.8)	442 (79.2)	3.76 (2.76-5.12)	<0.001*	3.09 (2.04-4.67)	<0.001*
	No	77 (6.5)	1104 (93.5)	Ref.		Ref.	
Other family smoking	Don’t have	39 (7.0)	521 (93.0)	1.06 (0.65-1.72)	0.814	0.88 (0.47-1.66)	0.698
	Yes	154 (11.6)	1172 (88.4)	1.86 (1.25-2.76)	0.002*	1.03 (0.62-1.70)	0.915
	No	32 (6.6)	453 (93.4)	Ref.		Ref.	
People smoke in school	Yes	139 (13.0)	927 (87.0)	2.46 (1.83-3.30)	<0.001*	2.02 (1.39-2.95)	<0.001*
	No	74 (5.8)	1212 (94.2)	Ref.		Ref.	
Teacher smoking	Don’t know	12 (4.3)	269 (95.7)	0.91 (0.47-1.78)	0.780	0.59 (0.27-1.32)	0.197
	Yes	179 (13.3)	1167 (86.7)	3.13 (2.15-4.54)	<0.001*	1.54 (0.95-2.50)	0.083
	No	35 (4.7)	713 (95.3)	Ref.		Ref.	
Attitude sum score; mean (SD)		3.12 (1.78)	4.00 (1.62)	0.73 (0.67-0.79)	<0.001*	0.76 (0.69-0.85)	<0.001*
Knowledge sum score; mean (SD)		1.71 (1.01)	1.88 (0.97)	0.84 (0.74-0.96)	0.012*	1.02 (0.85-1.23)	0.828

SD: standard deviation; OR: odds ratio; AOR: adjusted odds ratio; CI: confidence interval.

## Discussion

The study demonstrated that the prevalence of any form of tobacco smoking among adolescents in Iraq was 30.3%, while E-cigarette smoking was 9.7%. Cigarettes were the main form of tobacco used, followed by cigars and shisha. The strongest predictors of tobacco and E-cigarette smoking among adolescents were male gender, siblings smoking, and people smoking at school. Adolescents with attitudes against smoking were at less risk of smoking tobacco or E-cigarettes, while more knowledgeable adolescents were at more risk of smoking tobacco smokers only.

Male adolescents were at almost double the risk of being tobacco or E-cigarette smokers compared to females. Previous study in Erbil city in Iraq indicated that the strongest predictors of cigarette smoking was male gender.^14^ Gender differences in smoking prevalence for tobacco and E-cigarettes can be attributed to societal and cultural values in Iraq. The Iraqi Kurdistan culture consider smoking as taboo for both genders but would turn a blind eye and tolerate male smoking compared to female smoking behaviors.^21-23^ This is due to deeply embedded patriarchal beliefs that places less judgement on men and their views and practices, while women are criticized and condemned for the same practices. This pattern is also seen in Arab countries, such as Syria, where the most common reason women reported not being smokers was traditions and social norms.^24^

Adolescents of low socioeconomic status were less likely to smoke tobacco or E-cigarettes, suggesting that adolescents with higher weekly spending money could be smokers. A study based in Iraq had similar findings and proposed that access to spending money could help adolescents obtain cigarettes.^23^ Along with access to money, lack or poor enforcement of banning tobacco sale to minors makes it easy for adolescents to obtain tobacco products.^21^

Siblings have an even stronger influence on adolescent tobacco and E-cigarette smoking habits compared to parents; adolescents with smoking siblings were found to be about three times more likely to smoke tobacco or E-cigarettes. A 2021 study in Saudi Arabia demonstrated that adolescents who have smoking siblings had seven times more risk to smoke tobacco, and six more the risk of tobacco smoking intension.^25^ The strong association between adolescents smoking frequency and siblings influence could be attributed to the shared environment and social connectedness that siblings share.^26^ Thus, adolescents who live in and share the same environment as their siblings are more likely to acquire similar habits, and in turn picking up similar smoking habits as their siblings.

Exposure to parental and teacher smoking proved to increase risk of adolescent tobacco and E-cigarette smoking. Two studies, conducted in Lebanon^27^ and Morocco,^28^ reported increased risk of adolescent tobacco smoking with exposure to parental tobacco smoking. The research conducted using the GSHS survey in the Eastern Mediterranean Region reported a significant association between parental smoking (father, mother, or both) with cigarette smoking among teenagers with an AOR of 1.3 (95% CI 1.21-1.41).^15^ Teachers smoking in schools was also associated with higher risk of tobacco smoking among adolescents, as daily exposure to teachers’ smoking on school premises had higher impact on student smoking behaviors than having a parent who smokes.^27-30^ With the rate of teachers smoking in schools being higher than that of parents, it is imperative that policy-makers implement smoke-free schools in Iraq, reducing exposure and influence to smoking since the current 2012 anti-smoking law allows for schools to have a designated smoking area away from the sight of nonsmokers. Growing adolescents are influenced by people that they view as authoritative figures, such as parents and teachers, and attempt to imitate their behaviors.^8,31^ This observation can be explained by the Social Learning Theory, indicating that social behavior is acquired from observing and imitating the behaviors of role models.^31^ Negative attitudes notably decreased the likelihood of tobacco and E-cigarette smoking, yet knowledge plays a larger role in influencing tobacco use among adolescents. An Iraq-based study reported that adolescents with strongly negative attitudes towards E-cigarettes would consume them nonetheless.^21^ This is attributed to the lack of information and awareness of E-cigarette use in the community, as adolescents believed E-cigarettes impose less harm than traditional cigarettes.^21^ The lack of knowledge was positively associated with adolescents being more susceptible to E-cigarette usage. Despite that, due to the limited number of knowledge questions regarding E-cigarettes included in the study, it is difficult to draw concrete conclusions regarding the effect of knowledge on adolescents’ E-cigarette smoking habits.

The GYTS survey had a large, representative sample of adolescents attending schools in Iraq, and included diverse questions regarding different types of tobacco and E-cigarette use. This is the first study to our knowledge that investigated the parents’ and teachers’ smoking habits and their association with adolescents’ tobacco and E-cigarette use. Previous studies looked at the relationship but not the statistical association and impact on adolescents.

One of the study limitations was that adolescents’ tobacco and E-cigarette use was self-reported and that students might have under reported their smoking habits, specifically female students due to cultural taboo and societal disapproval of females smoking tobacco. The GYTS school survey is only limited to adolescents attending schools, overlooking students who are not enrolled in school due to poor socio-economic status post-war conflicts.^32^ Children and adolescents are dropping out of schools and engaging in child labor to support their families, putting education at a lower priority.^32^ The effects of these circumstances on adolescents’ smoking habits need to be further investigated. Another limitation was the use of data dating back to 2019 while attending the rapidly changing societal context, particularly concerning the strategies of tobacco companies and the prevalence of tobacco prevention and control measures.

Additionally, the study is a secondary analysis of preexisting data from the WHO GYTS, therefore could not assess the effect of stressors and mental health factors of adolescents on tobacco and E-cigarette use. Another limitation is that the survey included a small number of attitude and knowledge questions limited to tobacco smoking only and few on shisha smoking, but not including other types of tobacco or E-cigarettes. The study could not assess if family, friends, or teachers aid adolescents in obtaining tobacco products; it would have given more insight of family and friends involvement in enabling adolescents to consume tobacco. Moreover, the cross-sectional nature of the study design doesn’t allow for inferring causality.

## Conclusion

In conclusion, one in three Iraqi adolescents attending school reported using tobacco, while one in ten reported smoking E-cigarette smoking. Exposure to family and teachers smoking demonstrated significant associations with adolescent smoking. As such, policy makers should enforce stricter policies to ensure safer school environments that do not expose adolescents to smoking habits of teachers or other students. Furthermore, this study indicated a rise in tobacco smoking rates among adolescents from 21% in 2012 to 30% in 2019, which could be influenced by post-war conflicts, accompanied with psychological factors and nicotine addiction. Investigating post-war mental health effect on adolescents is worth looking at as well as investigating its effects on tobacco smoking and other health-related behaviors. There is no existing policy or law regulating public indoor E-cigarette use in Iraq. More studies, taking into consideration all above limitations, on E-cigarette use in Iraq and its prevalence can assist and directs policymakers to implement interventions that can help tackle the issue and reduce public indoor E-cigarette smoking in the future, especially in educational institutions and schools.

## Data Availability

The dataset and code book associated with the core GYTS questionnaire modules are made available to the public on the WHO and CDC websites. Data from core-expanded questions and country-specific questions are not made public. No school or student identifiers are included in the public use dataset (https://extranet.who.int/ncdsmicrodata/index.php/catalog/899#metadata-producers_sponsors).*
